# Periparturient stocking density affects lying and ruminating behavior and one-week-calf performance of Holstein cows

**DOI:** 10.5713/ajas.20.0126

**Published:** 2020-06-03

**Authors:** Mingming Jiang, Gibson Maswayi Alugongo, Jianxin Xiao, Congcong Li, Yulin Ma, Tingting Li, Zhijun Cao, Dasen Liu

**Affiliations:** 1 College of Animal Science and Technology, Northeast Agricultural University, Harbin 150030, China; 2 College of Animal Science and Technology, State Key Laboratory of Animal Nutrition, China Agricultural University, Beijing 100193, China; 3 College of Animal Science, Heilongjiang Agriculture Economics Vocational College, Mudanjiang 157041, China; 4 State Key Laboratory of Animal Nutrition, Institute of Animal Sciences, Chinese Academy of Agricultural Sciences, Beijing, 100193, China; 5 College of Animal Science and Technology, Xinjiang Agricultural University, Urumqi 830052, China

**Keywords:** Peripartum Dairy Cow, Stocking Density, Behavior, Productivity, Metabolism

## Abstract

**Objective:**

This study aimed to investigate the effect of stocking density on the behavior, productivity, and metabolism of periparturient Holstein cows as well as calf performance.

**Methods:**

A total of 48 periparturient cows were randomly assigned into three groups at 28 days (±3 days) before their expected calving date. The stocking densities of the groups, relative to the standard cubicle and feed bunk number, were i) 80% (13 cows), ii) 100% (16 cows), and iii) 120% (19 cows). Lying and rumination behavior was recorded using electronic data loggers and HR-Tags from d −21 (“d-” means days before calving) until the calving date, d 0. Lying time was assessed to determine the diurnal total hours spent lying per day. Rumination time was averaged in 2 hours interval periods over 24 hours during the experimental period.

**Results:**

Cows in the 80% group spent more time lying and ruminating between d −21 and d −7 and tended to ruminate more between d −14 and d 0. Calcium levels tended to be higher for cows in the 80% group, no other observable differences were found in monitored blood parameters. Moreover, 3.5% fat corrected milk and energy corrected milk yields were higher in 80% group in the first month of lactation. No other observable differences were found in the yield and composition of colostrum and milk in the first 10 months of lactation. The growth and performance of calves in the first week of life was not affected by stocking density of the dams.

**Conclusion:**

We concluded that lower stocking density may increase lying and ruminating behavior of prepartum Holstein cows. However, this did not translate into improved productivity and metabolism.

## INTRODUCTION

During the transition from gestation to lactation, dairy cows experience several social stressors including regrouping and nutritional changes. If these stressors are not managed well, they could exacerbate the incidences of metabolic diseases immediately after parturition in cows [1]. Overstocking is frequently practiced on large modern dairy farms with intensive farming systems [2]. High stocking density may escalate competition at the feed bunk, increase disease incidences and impact cow welfare [3], thus resulting in lower profit margins for farmers [2].

The recommended stocking density during the prepartum period is at least one cubicle of resting space per cow [4]. Various studies have observed that elevated stocking density (200% vs 100% cow to feed bunk) and feed bunk space (30 vs 60 cm/cow) may result in increased standing time, rate of feeding displacement, and feed sorting behavior, as well as decreased overall feed intake [5,6]. It is also reported that cows will typically occupy 80% of the feed bunk space during the peak feeding time after fresh feed delivery [7]. Since adequate feed intake is a pre-requisite for healthy post-partum cows, the observed behaviors are likely to affect post-parturition physiology. Monitored lying time has been suggested as a marker of cow comfort. Recently, a large scale study suggested that lying time along with energy and calcium (Ca) levels were critical for transition cows [8]. Notably, blood parameters, such as non-esterified fatty acids (NEFA), indicate the health of Holstein cows [8,9]. Additional controlled studies are needed to understand better the effects of stocking density stress in transition cows.

Prepartum management may not only affect the cow and its health, but it can also have detrimental effects on *in utero* calf development, with almost 60% of calf growth happening in the third trimester. Although studies on stocking density are on the increase, research regarding the *in utero* environment of cows are scarce. A few studies on nutrition and heat stress have indicated that calves are affected by pre-partum cow management [10]. It remains to be determined whether such deleterious effects can be extended to stocking density or not. This is because stocking density experiments are difficult to conduct owing to the dynamics of the sample size, especially around calving when cows are usually replaced to keep the original density [9,11]. Moreover, the influence of stocking density on cow behavioral patterns and the performance of their progeny is still unclear. The objective of this study was to determine whether the stocking density could impact the behavior, productivity and metabolism of prepartum Holstein cows or not, and to investigate the role stocking density plays on the colostrum quality as well as the performance of one week old calves.

## MATERIALS AND METHODS

### Animals, treatment and management

All the experimental protocols followed in this study were approved by the Institutional Animal Care and Use Committee of the China Agricultural University (Beijing, China). The experiment was conducted between December 2017 and January 2018. In total, 48 multiparous prepartum Holstein cows (dried off d 60 before expected calving date) were selected and randomly assigned into three pens (20 m long and 12.8 m width) at 4 weeks (d 28±3) before parturition. All the selected cows were entering the 2nd to the 4th lactation based on their proximity to their calving dates from a dry off group of 300 cows at Zhongdi Farm in Beijing, a commercial farm with 4,500 heads of dairy cattle. Each pen was assigned to one of the three treatments and balanced for parity (2.8±0.1), previous milk yield (37.0±6.2 kg/d), and body weight (BW, 797.6±71.3 kg). Cows were weighed on d −21 (“d-” means days before calving) and d 1 (“d” means days after calving) relative to parturition. Cows had a similar body condition score (BCS) of between 3 and 5 (average 3.25±0.25; 1 to 5 scale; 1 = emaciated, 5 = obese) and a locomotion score of under 2 (1 to 5 scale; 1 = normal gait, 5 = severely lame) at the time of enrollment [12]. In each pen, there were 16 cubicles (head to head water beds, 2.40 m long×1.20 m wide; Advanced Comfort Technology Inc., Sun Prairie, WI, USA) and 16 feed bunks (1.00 m wide× 0.75 m high×0.84 m depth; Roughage Intake Control System, Insentec B.V, Marknesse, the Netherlands).

The stocking density of the three treatments were calcu lated as the number of cows relative to the cubicle and feed bunk number, which were: i) 80% (13 cows), ii) 100% (16 cows), and iii) 120% (19 cows). One thermostatic water trough (366×56 cm) with two watering openings was available in each pen. The feed alleys and back alleys were 4.25 m and 3.70 m in width, respectively. The floor was scraped with an automatic scraper system (GEA Farm Technologies, Düsseldorf, Germany). The cubicles were fitted with waterbeds that were resupplied with approximately 10.0 cm of rice husk once per week. The cows were provided a close up diet (total mixed ration) feed and fresh cow diet (Table 1), pre- and post-parturitiion, respectively. The feed bunks were filled twice daily at 08:00 am and 15:00 pm for *ad libitum* intake. All cows underwent a daily clinical examination by the farm veterinarian throughout the study period. In addition, the pens were monitored *via* a video feed in proximity to the pens.

After calving, the cows were transferred to a separate pen for post-calving management and health examination. The cows received a Ca supplement (Bovikalc, Boehringer Ingelheim Vetmedica GmbH, Ingelheim am Rhein, Germany) immediately after calving and received the second Bovikalc 12 h later. Cows also received an oral administration of Propylene Glycol and Ca-P-Mg (Meilin calcium oral, 500 mL/bottle; Huataiyuan Pharmaceutical Co., Ltd, Beijing, China) mixture during the 12 h. The calf was moved within 5 minutes after calving to a newborn calf room. Cows deemed healthy one day post-calving were moved to a free-stall pen with 240 cubicles and 260 headlocks. Calves received 4 L of colostrum within 1 h of parturition (immunoglobulin G >22 g/dL) as per the farm protocol. Calves were transferred into individual hutches bedded with straw on d 1, just before the first milk feeding, where they were monitored for 7 days after birth. Calves were reared under identical conditions. They received 3 L of pasteurized waste milk twice daily at 7:00 am and 15:00 pm. BW was recorded immediately after birth and on d 7 using a digital scale. Average daily gain was calculated by dividing the difference between initial BW and final BW by number of days.

### Behavioral observation

Electronic data loggers (HOBO Pendant G, Onset Computer Corp., Bourne, MA, USA); validated by Ledgerwood et al [13] were placed on the right hind leg of prepartum cows at 22 days before expected date of parturition and removed immediately after calving to assess the lying time. The loggers were frequently checked to ensure they were well fastened on the leg. Data from individual animals were exported from the HOBO ware Pro software to an excel spreadsheet (Microsoft Corp., Redmond, WA, USA). Lying time was summarized to determine the diurnal total number of hours spent lying in a day. Rumination time was assessed with HR-Tags (SCR Engineers Ltd., Netanya, Israel) that were fitted to the left side of the neck of each cow from day of enrollment (d −28) to calving (d 0) of each experimental animal and averaged in 2 h interval periods over 24 h.

### Colostrum and milk parameters

After calving, colostrum yield (the first time milking after calving) was recorded. The total protein (TP) of colostrum was tested by the PAL-1 type digital Brix refractometer (ATAGO, Bellevue, WA, USA) and pH was analyzed by a portable pH meter (LAQUA twin, Horiba Scientific, Edison, NJ, USA). Cows were milked thrice daily. Milk yield of each cow was collected on day 15 and every 15th day of the month thereafter, post-calving and averaged as the daily milk yield. Subsequently, milk samples were collected from three time milking points and then mixed (4:3:3, composite from each daily milking) for the analysis of milk composition (Dairy Products Quality Supervision and Inspection Center, Beijing, China) such as fat, protein, lactose, and urea nitrogen using a Combi Foss FT+ instrument (Foss Electric, Hillerød, Denmark). Somatic cell counts (SCC) were analyzed by a Fossomatic 5000 apparatus (Foss Electric, Denmark). Fat-corrected milk yield (FCM) was calculated using the equation: 0.432×milk yield (kg/d)+16.23×milk fat yield (kg/d); and the energy corrected milk yield (ECM) was calculated based on the following equation: 0.327×milk yield (kg/d)+12.95× milk fat yield (kg/d)+7.2×milk protein yield (kg/d).

### Blood parameters

Blood samples were collected from the coccygeal vein on d −7, 0, 1, and 7 relative to the expected calving date for all cows and on d 0, 1, and 7 of age from the jugular vein for the calves. All blood samples were collected into vacuum blood collection tubes (Vacutainer, Becton Dickinson, Franklin Lakes, NJ, USA) containing no anticoagulant. The blood sampling time for both dairy cows and calves was approximately 6:00 am before the morning feeding, except for d 0 which was sampled right after calving and before colostrum feeding for cows and calves, respectively. The blood was centrifuged at 2,400×g for 15 min at 4°C. The serum was stored at −20°C in a freezer until analysis.

The samples were analyzed using commercially available kits according to manufacturer’s instructions. The concentration of cortisol (COR), total cholesterol (TC), triglycerides (TG), TP, NEFA, β-hydroxybutyrate (BHBA), blood urea nitrogen (BUN), and Ca were determined in the serum. All blood samples were quantified by an automated biochemistry analyzer (Gaomi Caihong Co Ltd, Shandong, China) except for COR that was measured by a multichannel radioimmune-counter (Zhongcheng Mechanical and Electrical Technology Co Ltd, Hefei, China). In calves, COR, BUN, and TP were determined, with TP being measured by a handheld optical refractometer.

### Rumen fermentation parameters

Rumen fluid was collected using an oral stomach tubing 4 h after cows had had their morning feed on d −15, −7, and 7 relative to parturition. The first (150 mL) was discarded to avoid saliva contamination. The rumen pH level was immediately measured, and 50 mL of each sample was immediately stored at −20°C for further analysis. Samples were analyzed for total volatile fatty acids (VFA), proportions of acetate, propionate, butyrate. valerate, isobutyrate, isovalerate, and ammonia.

### Statistical analysis

Ten cows per treatment were chosen for analysis purposes. All statistical analyses were performed using MIXED procedure of SAS (SAS Institute Inc., Cary, NC, USA). Continuous variables with repeated measurements including lying and ruminating behavior, the plasma and ruminal fermentation parameters were analyzed with the fixed effects of treatment, day of measurement, interaction between treatment and day of measurement, and the random effect of cow or calf nested within treatment. Variables with a single measurement during the study, such as milk production and composition, average calving date and colostrum quality were analyzed with the fixed effects of treatment of cow and the random effect of cow nested within treatment. It should be noted that feed intake data is excluded due to the technical issues experienced with the feeder. All treatment results are reported as least squares means. Significant differences were declared at p≤0.05 and tendencies at p≤0.10.

## RESULTS

### Feeding and behavior

**Figure 1:**
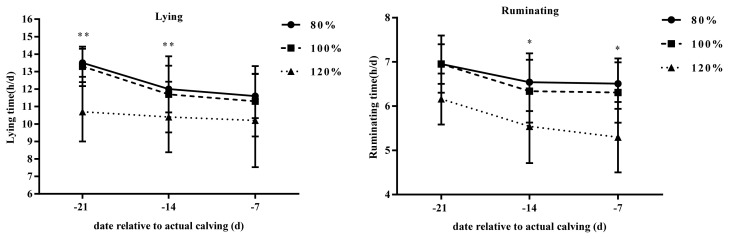
Effect of different feeding density on lying time and ruminating time. During the prepartum period (d −21 to −7 before calving). Lying and rumination behavior was recorded using electronic data loggers and HR-Tags from. Lying time was assessed to determine the diurnal total hours spent lying per day. Rumination time was averaged in 2 hours interval periods over 24 hours during the experimental period. Different periparturient stocking density is indicated by circles (80%), squares (100%), and triangles (120%). Asterisks denote groups containing differences, ** Means a significant effect (p<0.05), * means a tendency of effect (p<0.10).

The behavioral results of lying and ruminating are reported in Table 2 and Figure 1. Cows in the 80% stocking density group spent more time lying (p<0.05) and ruminating (p< 0.03) compared to cows in both the 100% and 120% groups. This finding was more apparent on d −21 and d −14 relative to parturition. Ruminating time tended to be significantly different on d −14 and d −7 relative to parturition (p<0.10).

### Colostrum and milk

The average yield, composition and quality of colostrum and milk are listed in Table 3 and 4. No differences were observed in the overall colostrum and milk yield, colostrum (TP), and milk (milk fat, milk protein, milk urea nitrogen and lactose) composition and quality (SCC and pH). However, a significant effect was observed for milk yield, FCM and ECM (p = 0.01) in the first month of lactation (data not shown) with the 80% group cows having greater yields.

### Blood parameters

**Figure 2:**
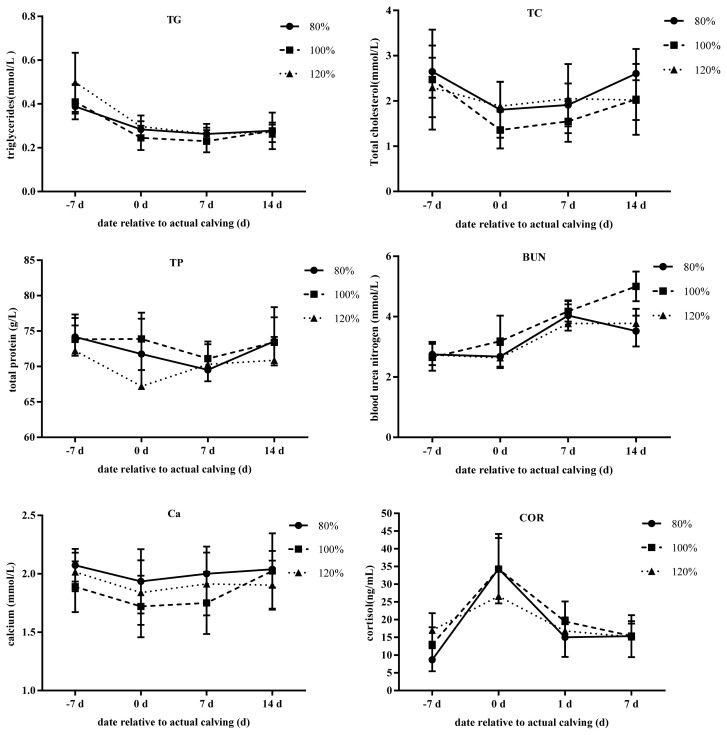
Effect of different stocking densities on blood parameters of transition cows. Blood samples were collected from the coccygeal vein on d −7, 0, 1 and 7 relative to the expected calving date for all cows. All blood samples were collected into vacuum blood collection tubes. Different periparturient stocking density is indicated by circles (80%), squares (100%), and triangles (120%). TG, triglycerides; TC, total cholesterol, TP, total protein; BUN, blood urea nitrogen; Ca, calcium; COR, cortisol.

**Figure 3:**
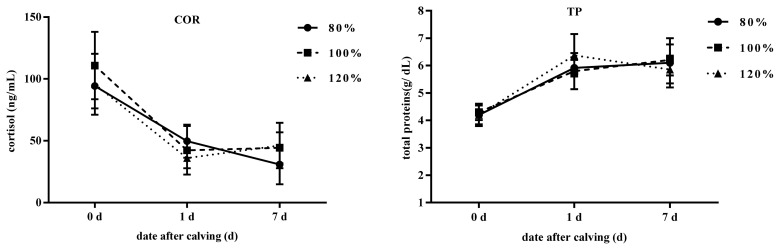
Effect of different stocking densities on blood parameters of newborn calves. Blood samples were collected from the jugular vein on d 0, 1, and 7 of age for the calves. All blood samples were collected into vacuum blood collection tubes containing no anticoagulant. Different periparturient stocking density is indicated by circles (80%), squares (100%), and triangles (120%). COR, cortisol; TP, total protein.

The effect of stocking density on blood parameters in transition cows (Table 5; Figure 2) and calves (Table 6; Figure 3) are reported. The stocking density was found to relate to Ca levels (p = 0.07), but no other observable effect on the blood parameters was measured. The COR, TC, TG, TP, BUN, and Ca were affected by time (p<0.05). Meanwhile, treatment× time interactions were absent in these parameters, except for a trend in TG (p<0.09) and NEFA (p<0.08). These observations could be attributed to higher concentrations in TG on d −7 in the 120% group and lower concentrations of NEFA on d 0 for the 80% group. No time effect was observed in NEFA and BHBA in this study. In calves, COR, BUN, Ca, and TP were not different between groups at any of the days sampled. However, COR and TP changed with time (p<0.05).

### Rumen fermentation parameters

**Figure 4:**
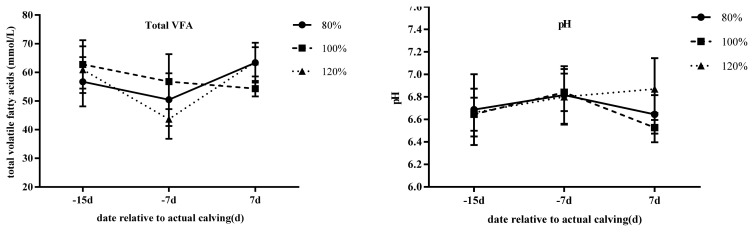
Effect of different stocking densities on total volatile fatty acid (TVFA) and pH of transition cows. Rumen fluid was collected using an oral stomach tubing 4 h after cows had had their morning feed on d −15, −7, and 7 relative to parturition. Different periparturient stocking density is indicated by circles (80%), squares (100%), and triangles (120%).

The proportions of total VFA, acetate, propionate, butyrate, valerate, Isobutyrate, and isovalerate concentrations as well as acetate/propionate ratio, ammonia nitrogen and pH are reported in Table 7 and Figure 4. There were no treatment or treatment by time effects detected in this trial. Only acetate, valarate, isobutyrate, isovalarate, and ammonia nitrogen changed with time (p<0.05). Concentration of acetate and ammonia nitrogen decreased as the cows approached parturition, while, valerate, isobutyrate, and isovalarate increased. Post-parturition the reverse happened. Ruminal pH was also similar between groups but changed with time (p<0.01).

### Body weight and structural measurements for calves

The BW, body length, withers height, breast circumference and abdominal circumference are shown in Table 8. There were no differences between d 1 and 7 after birth in all the parameters, but the calves increased in BW during this time period (p<0.01).

## DISCUSSION

### Behavioral measures

In this study, the results indicated that lying and rumination behavior can be affected by stocking density in the last 21 days of pregnancy of Holstein cows. Specifically, understocked cows (80% group) spent more time lying and ruminating compared to the other two groups (100% and 120% groups). This was more evident from d −21 to d −7. However, the lying time was similar at d −7 to d 0. These results are in agreement with a previous study [14], which concluded that cows kept in fewer stalls (high stocking density, 150%) spent less time lying down compared to the control group (stocking density of 100%). We observed a general decline in lying time among all cows as they approached parturition. Cows tend to be more anxious as they near parturition, hence they will spend more time standing [15]. The average lying times recorded were within established ranges reported in literature. It has also been suggested that parity has an effect on the social behavior of cows [1], hence we selected cows of ≥2 parity, which may have limited greater variation among groups.

Increased stocking density can also result in competition for available space [16] due to allelomimetic behavior, which results in limited time spent lying and eating for some animals [17]. Rumination time tends to decrease as cows approach calving. Increased rumination could be linked to higher resting time reported in this study though this has not been confirmed experimentally [18]. However, our findings demonstrated that rumination was not influenced by the stocking density. More research with larger sample sizes will help clarify the relationship between stocking density, lying time and rumination.

### Colostrum and milk yield and quality

Higher stocking density before parturition has been linked with decreased milk yield in the first 85 days of lactation [19]. However, most studies have reported no difference in milk yield in response to stocking densities before calving [20]. For example, a study on prepartum Holstein cows with two treatments of 80% cows to stalls and 90 cm of feeding space per cow (understocked) and 120% stocking density and 45 cm of feeding space per cow (overstocked), found no difference in the milk yield and composition for the first 5 weeks postpartum [11]. In our study, we found that milk yield, 3.5% FCM and ECM was greater for cows in 80% stocking density in the first month than that in 100% and 120% stocking density. The different stocking density during the prepartum period had no effect on the yield and quality of milk from the second month of lactation.

### Blood parameters in cows and calves

During the transition from late gestation to lactation, changes in the endocrinal system as well as feed intake are expected, which can affect metabolic status. By around 10 days before parturition, the NEFA concentration begins to rise in the blood irrespective of the decrease in dry matter intake [21]. In an all-in-all-out entry of cows, where agonistic behavior may result in reduced feed intake compared to traditional weekly entry, NEFA and BHBA concentrations did not differ between groups [22]. The lack of change in NEFA and BHBA levels is notable, although some previous research has shown that these metabolites can remain unchanged until very close to calving date [20]. Hormone concentrations that support gluconeogenesis and adipose tissue mobilization change in an effort to provide enough energy for the fetus and the developing mammary gland [23,24]. Lack of change in the blood parameters suggests similar energy status between the experimental groups, while changes in NEFA concentrations could be attributed to the short window of sampling (d −7 to d 7). Both BHBA and TG are the end products of non-oxidized fatty acids. Concentrations of TG tended towards significance, which is related to treatment × time. Increased TG is associated with higher concentrations of NEFA in the serum, and may accumulate in liver when greater amounts of NEFA are released from the adipose tissue [24]. However, thresholds at which TG begins to affect other hepatic processes are not clear [24]. We did not observe greater clinical signs in the cows included in this analysis, which may have contributed to the observed metabolic changes. The serum concentrations recorded in this study were lower compared to a previous study conducted in the same facility [25] as well as experiments with similar low protein diets prepartum [26,27].

Under non-inflammatory conditions Ca is under hor monal control [24]. We found that the understocked (80%) cows tended to have higher blood Ca levels compared to the other groups (100% and 120%). Other studies have also found that cows that were stocked at 120% had lower Ca compared to those stocked at 80% [17]. For periparturient dairy cows at the onset of lactation, there is a large increase in mineral losses due to the high mineral requirement for lactogenesis and colostrogenesis and this could result in a decrease in blood Ca concentration. Therefore, we conclude that the 80% cows might have slightly better Ca homeostatic mechanisms which retained higher blood Ca level than the other groups. The COR is an important marker of stress in animals and tends to increase on the day of parturition. Previous studies have reported no effect on COR concentrations in blood or hair for cows under different stocking densities [28]. Late gestation metabolic stress is likely to affect the immune function, susceptibility to diseases and growth rates of dairy calves [10]. A recent review, concluded that, as long as feed, water and resting space are sufficient and competition is minimized, the immune function, metabolic status, and performance may not be negatively affected [29]. Although, feed and water intake were not measured in this experiment, the differences in lying time may not be significant enough to induce immunological and metabolic disruption resulting in differential measures and health issues.

For successful passive immunity in calves, the serum TP should exceed 5.2 g/dL 24 h after colostrum feeding. All calves had greater than 5.4 g/dL of TP, indicative of successful passive transfer of immunity. The COR is usually high in newborn calves, and decreases with age as observed in this study. The Ca and BUN were within concentrations reported elsewhere for 1 week old calves.

### Calf growth parameters

The BW and structural measurements of calves were also similar between treatments in this study. Similar BW of new born calves were reported in another study of cows under different stocking densities simulated by introducing new animals to the experimental group [28]. The calves had increased BW by 1 kg at d 7. Growth rates in calves can be expected to be low in this period. Similar to BW there were no differences in the other body measurements between groups.

### Rumen fermentation parameters

The VFA concentration and pH level were not different between groups at the selected days of sampling. However, the proportion of acetate, valerate, isobutyrate isovalerate changed with time. During the transition period, farmers often change animal diets from dry-off to a fresh diet. In our experiment, cows were introduced to a lactating diet which is high in concentrates compared to the dry-off diet. Hence, the observed decrease in acetate could be associated with the change in diet from a high to low-fiber diet. The observed levels in total VFA is likely a response to greater organic matter available for rumen fermentation [30]. However, as in most other parameters investigated in this trial, no differences were observed between treatments.

## CONCLUSION

This study demonstrated that prepartum stocking density affected the lying and ruminating time from d −21 to d −7 relative to parturition. Cows in the 80% stocking density group were associated with greater lying and ruminating times compared to the other groups. However, the differences were not significant enough to result in changes in the metabolic status a week before and after parturition. Furthermore, calf growth and performance were not affected in the first week of life.

## Figures and Tables

**Table 1:** Ingredient composition and nutrient levels of experimental diets (dry matter, %)

Item	Close-up	Fresh cow diet
Ingredient		
Oat hay	23.72	-
Domestic alfalfa hay	-	15.99
Imported alfalfa hay	3.38	4.13
whole corn silage	38.63	21.15
Ground corn	4.85	1.95
Corn-steam flaked	-	20.73
Extruded soybean meal	1.67	2.15
Soybean meal	9.87	11.62
Palm kernel meal	1.32	-
Soybean hulls	6.23	11.66
Whole cottonseed	3.28	4.00
Molasses	1.99	-
Premix1)	5.06	6.62
Total	100.00	100.00
Chemical composition		
CP	11.89	15.42
Crude fat	3.51	4.85
NDF	38.53	31.02
ADF	23.00	20.11
Crude ash	7.60	7.63
Chloride	0.64	0.50
Calcium	0.63	0.92
Phosphorus	0.31	0.41
Magnesium	0.33	0.30
Sodium	0.31	0.44
Potassium	0.99	1.47
Sulfur	0.11	0.14

CP, crude protein; NDF, neutral detergent fiber; ADF, acid detergent fiber.

1) The premix provided to dry dairy cow per kg included: Vitamin A 770,000 IU, Vitamin D_3_ 192,500 IU, Vitamin E 7,000 IU, Cu 500 mg, Mn 1,800 mg, Zn 3,000 mg, I 56 mg, Se 19 mg, and Co 64 mg.

The premix provided to fresh cow per kg included: Vitamin A 116,500 IU, Vitamin D_3_ 57,000 IU, Vitamin E 750 IU, Fe 1.1 mg, Cu 0.7 mg, Mn 2.2 mg, I 76 mg, Se 5.5 mg and Co 29 mg.

**Table 2:** Effect of different stocking densities on lying and ruminating time of studied cows during 21 days before calving

Item	Stocking density (%)			SEM	p-value1)		
	
	80	100	120		Trt	Time	T×T
Lying time (h/d)	12.5^a^	11.3^b^	11.0^b^	0.39	0.05	<0.01	0.70
Ruminating (h/d)	6.68^a^	6.14^b^	6.11^b^	0.16	0.03	<0.01	0.95

Statistical analysis were carried out using MIXED procedure of SAS (SAS Institute Inc., Cary, NC, USA).

SEM, standard error of mean; Trt, treatment; T×T, treatment by time interaction.

1) p<0.05 means a significant effect; p<0.10 means a tended effect.

a,b Values within same row with different superscripts mean significant difference (p<0.05).

**Table 3:** Effect of different stocking densities on milk production and composition

Item	Stocking density (%)			SEM	p-value1)		
	
	80	100	120		Trt	Time	T×T
Milk yield	27.2	26.3	26.8	1.42	0.91	<0.01	0.47
3.5% FCM2)	28.8	26.7	26.6	1.38	0.47	<0.01	0.36
ECM3)	29.5	27.5	27.8	1.42	0.58	<0.01	0.39
Fat (%)	3.86	3.72	3.48	0.12	0.06	<0.01	0.08
Protein (%)	3.59	3.63	3.61	0.48	0.84	<0.01	<0.01
Lactose (%)	5.00	4.92	4.97	0.43	0.39	<0.01	0.45
Mun (mg/dL)	15.3	14.9	14.9	0.37	0.71	<0.01	0.20
SCC (1,000/mL)	216.1	290.1	272.0	69.8	0.75	0.01	0.97

SEM, standard error of mean; Trt, treatment; T×T, Treatment by time interaction; FCM, fat corrected milk; ECM, energy corrected milk; SCC, somatic cell count.

1) p<0.05 means a significant effect; p<0.10 means a tendency of effect (MIXED procedure of SAS [SAS Institute Inc., Cary, NC, USA]).

2) 3.5% FCM = 0.432×milk yield (kg/d)+16.23×milk fat yield (kg/d).

3) ECM = 0.327×milk yield (kg/d)+12.95×milk fat yield (kg/d)+7.2×milk protein yield (kg/d).

**Table 4:** Effect of different stocking densities on average calving date and colostrum quality and volume

Item	Stocking density (%)			SEM	p-value
	
	80	100	120		Trt
ACD	280.1	282.8	281.4	1.0	0.16
Brix	25.4	27.1	27.2	1.1	0.50
Weight	5.13	4.09	5.65	1.1	0.56

SEM, standard error of mean; Trt, treatment; ACD, average calving date.

**Table 5:** Effect of different stocking densities on blood parameters of transition cows

Item	Stocking density (%)			SEM	p-value1)		
	
	80	100	120		Trt	Time	T×T
NEFA (μmol/L)	62.6	67.9	70.8	5.1	0.55	0.86	0.08
BHBA (mmol/L)	0.56	0.60	0.58	0.54	0.86	0.86	0.33
TG (mmol/L)	0.31	0.29	0.33	0.02	0.20	<0.01	0.09
TC (mmol/L)	2.29	2.05	2.13	0.19	0.68	<0.01	0.21
TP (g/L)	72.2	72.6	70.1	1.1	0.23	0.02	0.31
BUN (mmol/L)	3.25	3.74	3.23	0.32	0.44	<0.01	0.86
Ca (mmol/L)	2.01	1.85	1.92	0.46	0.07	0.05	0.61
COR (ng/mL)	18.6	20.3	19.1	2.1	0.85	<0.01	0.55

SEM, standard error of mean; Trt, treatment; T×T, treatment by time interaction; NEFA, non-esterified fatty acids, BHBA, β-hydroxybutyric acid; TG, triglycerides; TC, total cholesterol; TP, total protein; BUN, blood urea nitrogen; Ca, calcium; COR, cortisol.

1) p<0.05 means a significant effect; p<0.10 means a tendency of effect (MIXED procedure of SAS [SAS Institute Inc., Cary, NC, USA]).

**Table 6:** Effect of different stocking densities on blood parameters of newborn calves

Item	Stocking density (%)			SEM	p-value1)		
	
	80	100	120		Trt	Time	T×T
COR (ng/Ml)	58.3	65.8	61.0	3.8	0.35	<0.01	0.28
BUN, (mmol/L)	3.14	2.89	3.20	0.24	0.59	0.22	0.48
Ca, (mmol/L)	1.88	1.99	2.12	0.81	0.15	0.51	0.54
TP (g/dL)	5.43	5.41	5.45	0.14	0.97	<0.01	0.24

SEM, standard error of mean; Trt, treatment; T×T, treatment by time interaction; COR, cortisol; BUN, blood urea nitrogen; Ca, calcium; TP, total protein.

1) p<0.05 means a significant effect; p<0.10 means a tendency of effect (MIXED procedure of SAS [SAS Institute Inc., Cary, NC, USA]).

**Table 7:** Effect of different stocking densities on ruminal fermentation parameters in cows before and after calving

Item	Stocking density (%)			SEM	p-value1)		
	
	80	100	120		Trt	Time	T×T
Total VFA (mmol/L)	99.53	100.08	99.82	2.86	0.87	0.10	0.56
Acetate proportion (%)	58.3	57.7	57.3	1.61	0.90	0.03	0.83
Propionate proportion (%)	23.4	25.5	25.2	1.2	0.45	0.90	0.77
Butyrate proportion (%)	9.17	9.45	9.18	0.50	0.90	0.16	0.34
Valerate proportion (%)	2.11	1.8	1.91	0.11	0.35	0.02	0.54
IsoButyrate proportion (%)	2.66	2.69	2.52	0.46	0.95	0.01	0.42
IsoValerate proportion (%)	3.89	2.94	3.71	0.64	0.55	0.01	0.45
Acetate/propionate	5.12	4.9	4.78	0.15	0.71	0.55	0.49
Ammonia nitrogen	10.92	12.32	11.76	0.99	0.60	0.01	0.98
pH	6.71	6.67	6.77	0.05	0.33	<0.01	0.27

SEM, standard error of mean; Trt, treatment; T×T, treatment by time interaction; VFA, volatile fatty acid.

1) p<0.05 means a significant effect; p<0.10 means a tendency of effect (MIXED procedure of SAS [SAS Institute Inc., Cary, NC, USA]).

**Table 8:** Effect of different stocking densities on birth body weight and structural measurement

Item	Stocking density (%)			SEM	p-value1)		
	
	80	100	120		Trt	Time	T×T
Body weight (kg)	39.6	42.5	40.5	1.3	0.30	<0.01	0.56
d 1	39.0	41.2	40.0	1.8	0.59	-	-
d 7	40.4	43.6	41.0	1.2	0.15	-	-
Body length (cm)	73.1	72.8	71.1	1.0	0.30	0.38	0.80
d 1	72.7	72.1	71.1	1.5	0.74	-	-
d 7	73.6	73.3	71.1	1.0	0.18	-	-
Withers height (cm)	76.8	76.5	77.3	0.9	0.75	0.43	0.47
d 1	76.1	76.9	76.6	1.2	0.90	-	-
d 7	77.6	76.1	77.9	1.1	0.45	-	-
Breast circumference (cm)	78.5	79.6	78.2	1.0	0.47	0.35	0.94
d 1	78.6	80.2	78.8	1.3	0.64	-	-
d 7	78.3	79.1	77.5	1.2	0.66	-	-
Abdomen circumference (cm)	80.2	80.8	79.9	1.0	0.72	0.30	0.67
d 1	80.1	81.9	80.6	1.4	0.65	-	-
d 7	80.3	79.8	79.2	1.2	0.80	-	-

SEM, standard error of mean; Trt, treatment; T×T, treatment by time interaction.

1) p<0.05, means a significant effect; p<0.10 means a tended effect (MIXED procedure of SAS [SAS Institute Inc., Cary, NC, USA]).
